# Targeting of Painting of fourth to *roX1* and *roX2* Proximal Sites Suggests Evolutionary Links Between Dosage Compensation and the Regulation of the fourth Chromosome in *Drosophila melanogaster*

**DOI:** 10.1534/g3.113.006866

**Published:** 2013-08-01

**Authors:** Lina E. Lundberg, Maria Kim, Anna-Mia Johansson, Marie-Line Faucillion, Rafael Josupeit, Jan Larsson

**Affiliations:** Department of Molecular Biology, Umeå University, SE-90187 Umeå, Sweden

**Keywords:** Painting of fourth, dosage compensation, heterochromatin, epigenetics, *Drosophila melanogaster*

## Abstract

In *Drosophila melanogaster*, two chromosome-specific targeting and regulatory systems have been described. The male-specific lethal (MSL) complex supports dosage compensation by stimulating gene expression from the male X-chromosome, and the protein Painting of fourth (POF) specifically targets and stimulates expression from the heterochromatic 4^th^ chromosome. The targeting sites of both systems are well characterized, but the principles underlying the targeting mechanisms have remained elusive. Here we present an original observation, namely that POF specifically targets two loci on the X-chromosome, *PoX1* and *PoX2* (POF-on-X). *PoX1* and *PoX2* are located close to the *roX1* and *roX2* genes, which encode noncoding RNAs important for the correct targeting and spreading of the MSL-complex. We also found that the targeting of POF to *PoX1* and *PoX2* is largely dependent on *roX* expression and identified a high-affinity target region that ectopically recruits POF. The results presented support a model linking the MSL-complex to POF and dosage compensation to regulation of heterochromatin.

The evolution of sex chromosomes, for example the X and Y chromosome pairs found in mammals and flies, makes it more difficult for balanced gene expression to be achieved. Although some genes located on the X-chromosome are expressed in a sex-specific manner, most genes require equal expression in males and females (Vicoso and Bachtrog 2009; Stenberg and Larsson 2011). The gradual degeneration of the proto-Y chromosome causes an increasing requirement to equalize gene expression between a single X (in males) and two X-chromosomes (in females) and to balance this level of expression with that of the two sets of autosomal chromosomes (Oliver 2007; Vicoso and Bachtrog 2009; Prestel *et al.* 2010a; Stenberg and Larsson 2011). This requirement has led to the evolution of dosage-compensation mechanisms. Such mechanisms must equalize the transcriptional activities of the two X-chromosomes in the homogametic sex with that of the single X-chromosome in the heterogametic sex, as well as balancing the relative expression levels between sex chromosomes and autosomes (Gupta *et al.* 2006; Nguyen and Disteche 2006; Deng *et al.* 2011). In fruit flies, both these requirements are solved by a 2-fold increase in gene expression that is restricted to the single male X-chromosome. This 2-fold increase is the result of a combination of a general buffering effect exerted on monosomic regions or chromosomes (Stenberg *et al.* 2009; Zhang *et al.* 2010; Lundberg *et al.* 2012) and an increase in expression from the male X-chromosome mediated by the male-specific lethal (MSL) complex (Hamada *et al.* 2005; Deng *et al.* 2009; Prestel *et al.* 2010a; Stenberg and Larsson 2011). The MSL-complex consists of two partly redundant noncoding RNAs, *roX1* and *roX2*, together with at least five MSL proteins (MSL1, MSL2, MSL3, MLE, and MOF) that “paint” the dosage-compensated male X-chromosome. MOF mediates acetylation of H4 at lysine 16 (H4K16ac), and enrichment for H4K16ac on the male X-chromosome is believed to cause decondensation of the chromatin fiber, which at least partly explains the hypertranscription of this chromosome (Gelbart and Kuroda 2009; Prestel *et al.* 2010a). The MSL-complex not only recruits MOF to the male X-chromosome but also constrains the activation potential of MOF (Prestel *et al.* 2010b; Sun *et al.* 2013). The male specificity of the MSL-complex is accomplished by the MSL2 protein, which is only formed in males and is rate limiting for the formation of the complex (Kelley *et al.* 1995, 1997; Bashaw and Baker 1997).

Understanding how the MSL-complex correctly targets the ~1000 active genes on the male X-chromosome is a key challenge in this area of research; targeting is currently thought to be initiated by sequence-dependent binding of the complex to 100−200 nucleation sites on the X-chromosome, which are termed chromatin entry sites (CES) or high-affinity sites (HAS) (Alekseyenko *et al.* 2008; Straub *et al.* 2008, 2013). Two of the strongest nucleation sites are the *roX1* and *roX2* loci (Kelley *et al.* 1999). Targeting to nucleation sites is followed by spreading to neighboring genes; this is dependent on active transcription (Sass *et al.* 2003; Larschan *et al.* 2007), MSL-complex concentration (Dahlsveen *et al.* 2006), level of affinity (Straub *et al.* 2008; Lucchesi 2009), and probably also sequence composition (Philip *et al.* 2012).

However, the dosage compensation mediated by the MSL-complex is not the only chromosome-wide compensatory mechanism that has been described in *Drosophila*. There is also the protein Painting of fourth (POF), which in *Drosophila melanogaster* specifically targets the 4^th^ chromosome (Larsson *et al.* 2001; Johansson *et al.* 2007a, 2012). POF binds to nascent RNA from actively transcribed genes on the 4^th^ chromosome and increases levels of expression of these genes (Johansson *et al.* 2012). The compensation mediated by POF is sufficient to allow survival of haplo-4 flies (Johansson *et al.* 2007a). The specific targeting of POF to the 4^th^ chromosome, the evolutionary connections between the 4^th^ chromosome, the male X-chromosome and dosage compensation, and the stimulatory effect of POF on gene expression all support the hypothesis that POF originates from a dosage compensatory system (Larsson *et al.* 2001, 2004; Larsson and Meller 2006; Johansson *et al.* 2007a,b; Stenberg *et al.* 2009; Stenberg and Larsson 2011). Although dosage compensation has probably been required, and thus evolved, on a gene-by-gene or block-of-genes basis, the existence of systems like the MSL-complex that act across a whole chromosome argues that some of the functions were pre-existing and were recruited to compensate the X-chromosome as the proto-Y chromosome started to degenerate (Stenberg and Larsson 2011). The targeting mechanisms of these systems and the relationship between them are therefore important issues to study in order to understand chromosome evolution and how balanced genome expression is established.

Here we provide a further link between these two chromosome-wide targeting systems by exploring an original observation that POF targets two loci on the X-chromosome in a female-specific context. The targeting of these loci depends on *roX* expression, and by constructing transgenes and examining their effects we have identified a POF high-affinity recruitment element.

## Material and Methods

### Fly strains and genetic crosses

Flies were cultivated and crossed at 25º in vials containing potato mash-yeast-agar. *roX1^ex6^*, *roX1^SMC40^*, and *roX1^ex84A^* strains were obtained from Victoria Meller (Wayne State University, Detroit, MI) and are described in Deng and Meller (2009). *roX1 roX2* double-mutant males were selected as non-green fluorescent protein males from a *y w roX1^ex6^ Df(1)roX2^52^ P[w^+^4Δ4.3]*/ *FM7i, P[w^+mC^ ActGFP]JMR3* stock obtained from Yongkyu Park (New Jersey Medical School, Newark, NJ). *roX1 roX2* double-mutant females larvae were selected from the cross: *y w roX1^ex6^ Df(1)roX2^52^ / Binsincy*; *P[w^+^4Δ4.3]*/+ × *y w roX1^ex6^ Df(1)roX2^52^ / Dp(1;Y)B^s^ y^+^ v^+^*; *P[w^+^4Δ4.3]*/+. Overexpression of *roX1* and *roX2* was performed using *y w*; *[w^+^ hsp83:roX1]* and *y w*; *[w^+^ hsp83:roX2]34B*, respectively. Female larvae overexpressing *msl2* were selected from a *w*; *P[w^+^ hsp83:msl2] msl3/TM6B* stock obtained from Mitzi Kuroda (Harvard Medical School, Boston, MA). The *P[w^+^ CkIIβ gDNA]* transgenic line was obtained from Thomas Raabe (Universität Würzburg) and is described in Jauch *et al.* (2002). The duplications of the *PoX1* and *PoX2* loci used were *w^1118^*; *Dp(1;3)DC112*, *PBac[y^+mDint2^ w^+mC^ DC112]VK00033* (92 kb inserted at 3L:65B2 covering *PoX1*), *w^1118^*; *Dp(1;3)DC244*, *PBac[y^+mDint2^ w^+mC^ DC244]VK00033* (87 kb inserted at 3L:65B2 covering the three genes *Ck2β*, *Hsc70-3*, *CG1578* in *PoX2*) and *Dp(1;3)DC246*, *PBac[y^+mDint2^ w^+mC^ DC246]VK00033* (102 kb inserted at 3L:65B2 covering the two genes *SelG* and *CG1840* in *PoX2*) (Venken *et al.* 2009, 2010). To visualize Setdb1 on polytene chromosomes, we used the strain *Setdb1^3HA^* (Seum *et al.* 2007), which contains a gene encoding hemagglutinin-tagged Setdb1, obtained from Carole Seum (University of Geneva). *Oregon R* was used as the wild-type strain.

### Transgenic flies

To generate the *P[w^+^ SelG CG1840]* transgene, a genomic fragment was amplified with Long PCR Enzyme Mix (Thermo Scientific) using the primers 5′-GT**GGATCC**AAAAATGGTCTTGTTCCACA-3′, 5′-CG**GCGGCCGC**AATTTGGCGGAAGATTCAAA-3′ with *Oregon R* genomic DNA as a template. Thus, the fragment included both the *SelG* gene and the *CG1840* gene as well as 1673-bp fragment upstream of *SelG* transcription start and 2733 bp downstream of the annotated transcript stop position of *CG1840*. The polymerase chain reaction (PCR) product was cut with *Bam*HI/*Not*I and ligated into a *P[w^+^ attB]* vector. The latter, which is a *pBluescript*-based plasmid supplemented with an *attB* recombination site and a *mini-white* marker gene, was provided by M. Savitsky. Embryo microinjection into the *Bl9750* strain was performed by BestGene (Inc).

### RNA-fluorescent *in situ* hybridization (RNA-FISH) and immunostaining of polytene chromosomes

Immunostaining of polytene chromosomes was essentially as described previously (Johansson *et al.* 2012). We used primary antibodies against POF raised in rabbit, diluted 1:500 (Larsson *et al.* 2004), or raised in chicken, diluted 1:100 (Larsson *et al.* 2001); against HP1a (PRB291C, 1:400, Covance); and against HA (MMS 101R, 1:100, Covance, for detection of Setdb1.3HA). Goat or donkey antirabbit, antichicken, and antimouse conjugated with Alexa-Fluor555 or AlexaFluor488 (1:500; Molecular Probes) were used as secondary antibodies.

For RNA-FISH combined with immunostaining, salivary glands were fixed in 3.7% formaldehyde in phosphate-buffered saline (PBS), 0.3% Triton X-100 for 20 sec followed by 2−3 min in 50% acetic acid, 1% formaldehyde. Polytene chromosomes were squashed using high pressure treatment (Novikov *et al.* 2007), quick-frozen in liquid nitrogen, and stored in ethanol at −20° until required for use. The slides were rehydrated in an ethanol series (1 min each in 95%, 70%, and 30% ethanol), then incubated for 15 min in PBT (*i.e.*, PBS, 0.1% Triton X-100), and the chromosomes were fixed in 3.7% formaldehyde in PBT for 15 min. The slides were then washed for 3× 3 min in PBT followed by prehybridization at 42° for 3−4 hr in hybridization mixture (5× saline sodium citrate (SSC), 5× Denhardts solution, 500 μg/mL cold DNA, 250 μg/mL transfer RNA, and 50% formamide).

The slides were then hybridized using a digoxigenin labeled antisense RNA probe against *RE64691* mixed with hybridization mixture in a wet chamber at 42° overnight. After hybridization the slides were washed in 2× SSC at room temperature for 1 min followed by 2× 30 min in 50% formamide, 5× SSC, 10 mM dithiothreitol at 42°, 2× 30 min in 2× SSC at 42°, 1 hr in 0.1× SSC at room temperature, and finally 10 min in PBT at room temperature. The slides were transferred to blocking solution (PBS, 1% Roche blocking reagent) and incubated for 30 min at room temperature, then incubated for 1 hr at room temperature with primary antibody raised against POF (1:500 dilution) and sheep antidigoxigenin (0.4 μg/mL; Roche). The slides were then washed for 3× 2 minutes in PBS + 0.2% Tween-20 at room temperature. They were incubated in the secondary antibodies donkey antirabbit and donkey antisheep conjugated with AlexaFluor555 or AlexaFluor488 (Molecular Probes; diluted 1:500), together with DAPI (5 µg/mL), for 1 hr at room temperature. Finally, the slides were washed for 4× 5 min in PBS + 0.2% Tween-20 and mounted with Vectashield (Vector).

### Chromatin immunoprecipitation (ChIP) and microarray analysis

For the POF ChIP-chip experiment, we dissected salivary glands from third instar larvae. The ChIP experiment was conducted as previously described (Johansson *et al.* 2007a,b) using 3 µL of anti-POF (Larsson *et al.* 2004) for precipitation. The purified ChIP and input DNA samples were amplified using a WGA2 GenomePlex Complete whole-genome amplification kit (Sigma-Aldrich) according to the recommendations of the supplier, and the amplified DNA was purified with a QIAquick PCR purification kit (QIAGEN). To verify that no amplification bias affected the enrichment profiles, we analyzed the ChIP DNA/input DNA ratio before and after amplification by using real-time PCR as previously described (Johansson *et al.* 2007b).

For tiling array analysis, the amplified ChIP DNA samples were fragmented, labeled, and hybridized to an Affymetrix *Drosophila* Genome 2.0 array. The signal intensity data generated were analyzed with Affymetrix Tiling Analysis Software (v. 1.1.02), using a 200-bp bandwidth as a smoothing parameter and setting a constraint of perfect match only. The enrichment profiles were produced as ChIP DNA/input DNA ratios expressed on a log_2_ scale. The complete data set is available at http://www.ncbi.nlm.nih.gov/geo/ (accession: GSE45402). The occupancy profiles obtained were visualized and analyzed using Integrated Genome Browser (7.0.1) (Nicol *et al.* 2009).

### Gene identification

To verify the presence of a novel gene between the two genes *Mnt* and *Rala*, we sequenced the Expressed Sequence Tag clone *RE64691* obtained from the *Drosophila* Genomics Resource Centre. To determine the 5′- and 3′-ends of the putative novel gene, we isolated poly(A)^+^ RNA from adult females using Dynabeads Oligo (dT)_25_ magnetic beads (Invitrogen) as previously described (Svensson *et al.* 2003) and verified the end sequences using the SMART-RACE cDNA-amplification kit (Clontech) according to a protocol provided by the supplier. The primers used were: *RE64691* gene specific primer 1: 5′-CAGAAATCGAGTGACACACACAGG-3′, and primer 2: 5′-GCTAACATAAGCCCACATCCACAC-3′, the nested gene specific primer 1: 5′-ACTATAAGTCCCCCGTGATGACAG-3′ and the nested primer 2: 5′-GCAGATGGAGACGGAAAGAGTAGG-3′.

## Results

### POF binds to two loci, *PoX1* and *PoX2*, on the X-chromosome in females

**Figure 1:**
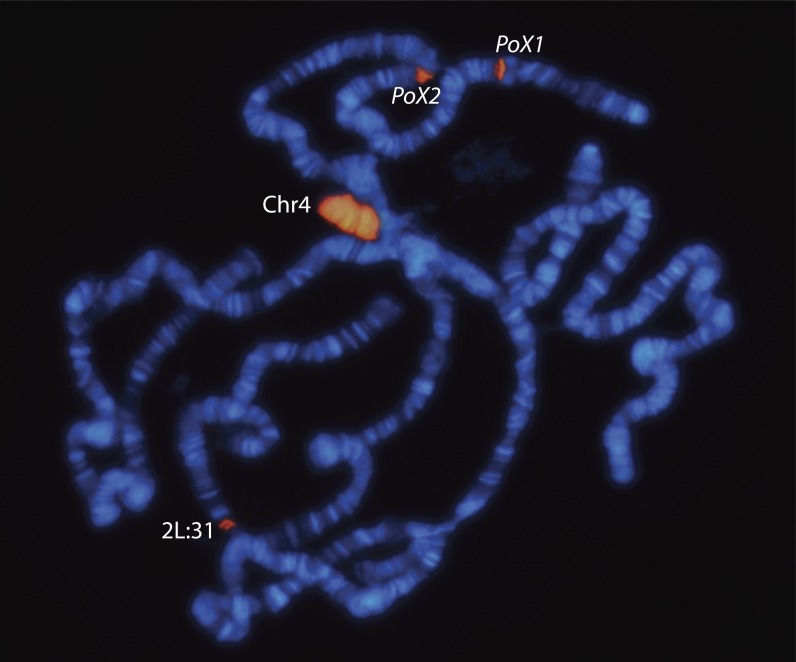
POF binds two sites on the X-chromosome in females only. Immunostaining of polytene chromosomes from a wild-type female. In addition to binding on the 4^th^ chromosome (Chr4), in this nucleus POF binds to two sites on the X-chromosome, *PoX1* and *PoX2* (seen in 30−50% of nuclei) and to region 2L:31 on the left arm of the second chromosome.

**Figure 2:**
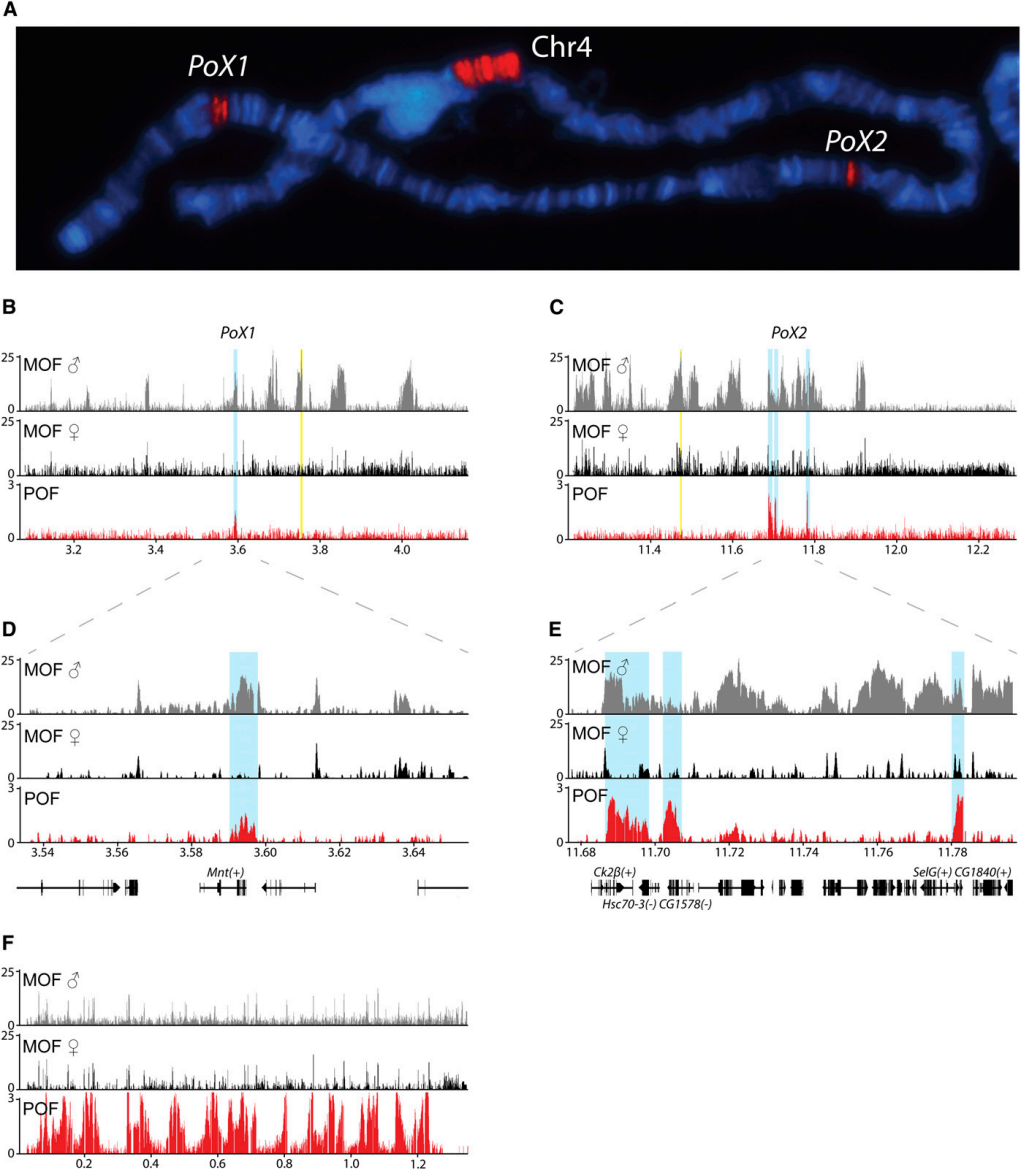
POF binding profiles of the X-chromosome regions including *PoX1* and *PoX2*. (A) Immunostaining of polytene chromosomes showing POF binding to the 4^th^ chromosome (Chr4) and the two loci *PoX1* and *PoX2* as indicated. (B) POF binding profile from salivary gland tissue ChIP-chip. An ~1-Mb region including the *PoX1* region (blue box) is shown. The binding profiles of MOF in males and females[from ChIP-seq (Conrad *et al.* 2012)] are shown for comparison. (C) POF binding profile in an ~1-Mb region including the *PoX2* regions (blue boxes). The plots show the mean enrichment ratios obtained using a bandwidth of 200 bp for the POF ChIP-chip. Numbers on the x-axis denote chromosomal position along the chromosome in Mb. The y-axis shows ChIP enrichments as log_2_ ratios. *PoX1* and *PoX2* are located downstream of genes *roX1* and *roX2* (indicated by yellow boxes), respectively. The *roX1* gene is transcribed from right to left and *roX2* is expressed in the opposite direction. (D and E) High resolution profiles of enrichment at the *PoX1* (D) and *PoX2* (E) sites. The genes bound by POF are indicated; genes expressed from left to right are indicated by (+) and genes expressed in the opposite direction are indicated by (−). (F) Profile of POF binding on the 4^th^ chromosome (~1.3 Mb) is shown for comparison.

We have previously shown that POF binds with high specificity to the 4^th^ chromosome in both males and females (Larsson *et al.* 2001; Johansson *et al.* 2007a,b; Figueiredo *et al.* 2012). With improved techniques for preparation and staining, we noticed that in good chromosome preparations from wild-type larvae two strong POF binding signals were visible on the X-chromosome. By sexing larvae we found that, out of the best 10−15 nuclei (fully polytenized and well spread) from each pair of salivary glands, 30−50% of all X-chromosomes in females showed one or two reproducible stained bands at cytological locations X:3E and X:10E−F (Figure 1). These bands were never detected in males. We named these loci *PoX1* and *PoX2* (POF-on-X) and found spreading of the POF binding in both loci (Figure 1 and Figure 2A). The presence and degree of spreading differed between nuclei, and we typically saw 1-3 bands decorated by POF in both *PoX1* and *PoX2*. In addition to the two loci on the X-chromosome, we occasionally observed POF binding in parts of the 2L:31 region in both males and females (Figure 1) (Lundberg *et al.* 2013).

### *PoX1* and *PoX2* correspond to specific genes on the X-chromosome

We were intrigued by the fact that the two loci, *PoX1* and *PoX2*, mapped cytologically close to *roX1* (X:3F) and *roX2* (X:10C) and decided to map these *PoX* loci more precisely by using ChIP-chip technology. We performed ChIP-chip analysis on chromatin extracts from salivary glands of 3^rd^ instar larvae. The ChIP-chip profiles generated confirmed the cytological observations (Figure 2). The *PoX1* locus corresponds to a strong binding peak/region ~200 kb downstream of *roX1* (Figure 2B). In this region, POF is enriched within the 3′-end of the *Mnt* gene and in a distinct 2-kb region between the genes *Mnt* and *Rala* (Figure 2D). The *PoX2* locus corresponds to three enriched regions covering the five genes *Ck2β*, *Hsc70-3*, *CG1578*, *SelG*, and *CG1840*, which are located ~200 kb downstream of *roX2* (Figure 2, C and E). To test whether POF binding enrichment was correlated with enrichment for the MSL-complex, we plotted MOF data generated from salivary glands of males and females, which was available from (Conrad *et al.* 2012). Some, but not all, genes in the *PoX1* and *PoX2* loci that were enriched in POF, also were enriched in MOF (and presumably the MSL-complex) in males. In females, MOF bound to the promoters of *Ck2β*, *SelG* and *CG1840* in the *PoX2* locus (Figure 2, D and E). We conclude that, with respect to pattern of binding to the *PoX1* and *PoX2* loci, there is no obvious link between POF and the MSL-complex that distinguishes the targeted genes from other X-linked genes.

### POF binds to a novel noncoding gene between *Mnt* and *Rala*

**Figure 3:**
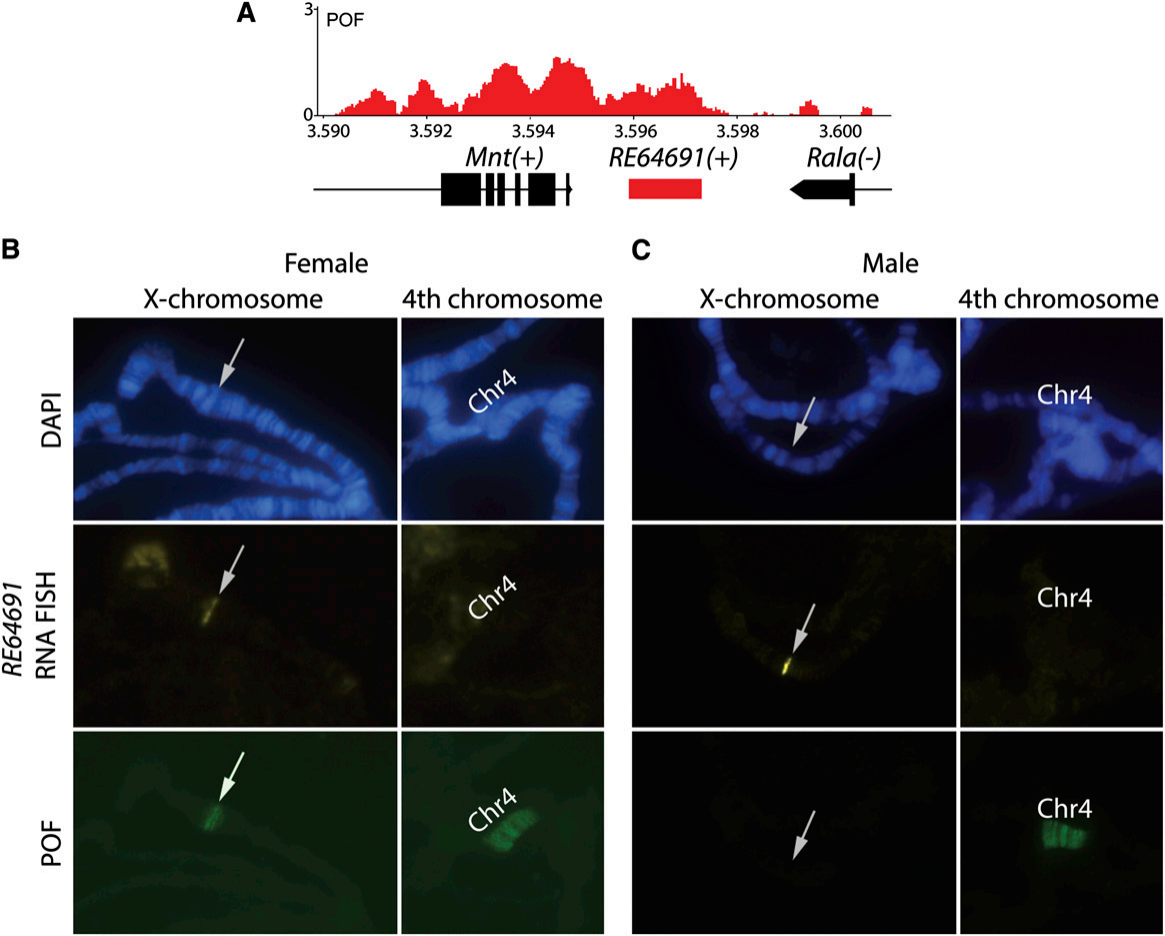
POF binds to a novel gene, *RE64691*, located between *Mnt* and *Rala*. (A) High-resolution enrichment profiling at the *PoX1* site shows that POF binds to a novel gene (indicated by a red rectangle) represented by the EST clone *RE64691*. (B and C) Immunostaining combined with RNA-FISH shows that the gene *RE64691* is expressed in both females (B) and males (C) but targeted by POF only in females (note that in contrast POF targets the 4^th^ chromosome in both males and females). The *RE64691* locus is indicated by an arrow. The tip of the X-chromosome and the 4^th^ chromosome from one representative female nucleus are shown in (B) and from one male in (C). The 4^th^ chromosome is indicated by “Chr4”.

In the *PoX1* locus, we found POF to be highly enriched within the 3′-end of *Mnt* and in a distinct region downstream of *Mnt* and *Rala*, which are transcribed in the opposite direction so that the region is downstream of both genes (Figure 3A). No gene had previously been annotated in this region, but a large number of expressed sequence tag (EST) cDNA clones are annotated according to the GBrowse genome browser at FlyBase (Tweedie *et al.* 2009). We sequenced one of these EST clones, *RE64691*, and confirmed using 5′- and 3′-RACE (*i.e.*, rapid amplification of cDNA ends) that this EST cDNA clone probably corresponds to a novel gene as illustrated in Figure 3A. The longest putative ORF is 34 amino acids and it is therefore possible that *RE64691* corresponds to a novel noncoding gene. Alternatively, *RE64691* belongs to the class of genes with short ORFs encoding small bioactive peptides (Frith *et al.* 2006; Kastenmayer *et al.* 2006; Hanada *et al.* 2007) as exemplified by, *e.g.* the *Drosophila* gene *tarsal-less* (Galindo *et al.* 2007; Kondo *et al.* 2010).

Anyway, intrigued by the possibility that POF targets a gene encoding a noncoding RNA (ncRNA), and bearing in mind the fact that ncRNAs have been shown to be important for correct targeting of the MSL-complex, we conducted RNA-FISH experiments to probe whether *RE64691* RNA was connected to POF targeting of chromosome 4. The RNA-FISH results showed that *RE64691* is expressed in salivary glands in both males and females, but no enrichment of *RE64691* RNA on the 4^th^ chromosome was detected (Figure 3, B and C). The detection of *RE64691* expression in both males and females indicated that expression *per se* could not explain the sex-specific targeting of *PoX1*. We therefore checked expression in salivary gland tissues of the five genes located in *PoX2* by using RNA-seq data from (Graveley *et al.* 2011). The five genes located in *PoX2* are expressed at similar levels in males compared to females (results not shown). We conclude that gene expression is not the determinant of the female specific targeting of POF to *PoX1* and *PoX2* and that *RE64691* RNA is not enriched in POF-bound regions outside the *RE64691* locus.

### POF and HP1a colocalize at *PoX* loci

**Figure 4:**
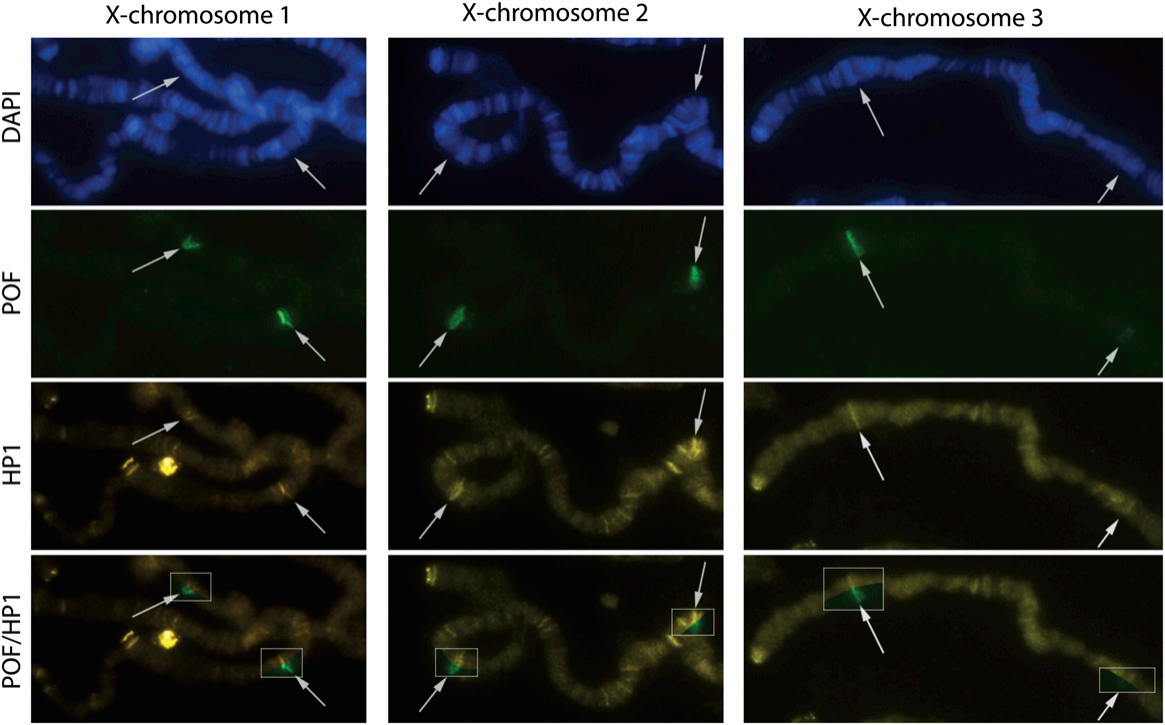
POF and HP1a binding overlap on the *PoX* loci. POF (green) and HP1a (yellow) on the X-chromosome in three representative female nuclei. The proximal parts of the X chromosomes are shown, and the *PoX1* and *PoX2* loci are indicated with arrows. The boxes in the POF/HP1 row show the combined image. Note that levels of HP1a binding at *PoX* loci are above the background staining level.

We have previously shown that POF and HP1a colocalize within the gene bodies of active genes on the 4^th^ chromosome (Johansson *et al.* 2007a,b; Figueiredo *et al.* 2012). Indeed, no POF binding has been detected without HP1a enrichment. We therefore asked whether the *PoX* sites, which are bound only in females and not in all individual nuclei, are also targeted by HP1a. We observed that in the cases where POF binds to *PoX1* and *PoX2*, weak but consistent enrichment for HP1a was present (Figure 4). We conclude that POF binding is accompanied by HP1a binding at all POF enriched sites including the female specific *PoX1* and *PoX2*.

### POF binding is dependent on *roX*

Several lines of evidence have linked POF to dosage compensation and indicated that it has an evolutionary relationship with the MSL-complex (reviewed by (Stenberg and Larsson 2011)). We were therefore fascinated by the close proximity of *PoX1* and *PoX2* to *roX1* and *roX2*, respectively. We asked whether the targeting by POF to *PoX1* and *PoX2* depended on *roX1* and/or *roX2*. When we analyzed the best 10−15 nuclei in each chromosome preparation (fully polytenized chromosomes with a well spread X-chromosome from one pair of glands), 30-50% of these nuclei showed *PoX1* and/or *PoX2* targeting by POF (Table 1). Using the same criteria, we found, to our surprise, almost no POF targeting to *PoX1* or *PoX2* in *roX1*- or *roX1* *roX2*-mutant female larvae (Table 1). Thus, the extent of POF targeting to *PoX* loci is dependent on *roX*; however, it does not require *roX* because in all *roX* mutant backgrounds tested we found individual nuclei (<5%) with clear targeting. Furthermore, overexpression of *roX2* (but not *roX1*) in females using *roX* transgenes under a *hsp83* promoter increased the targeting frequency. In contrast, expression of a partial MSL-complex in females decreased the targeting frequency (Table 1). We conclude that POF targeting to *PoX* loci in females is affected by but does not require *roX*.

**Table 1:** Frequency of *PoX1* and *PoX2* targeting by POF

Genotype	*PoX1* and *PoX2* Targeting	
	Female	Male
Wild type	+	−
*roX1^ex6^* or *roX1^SMC40A^* or *roX1^ex84A^*	+/−	−
*roX1^ex6^ roX2*	+/−	−
*y w*; *P[w^+^ hsp83:roX2]34B*	*++*	*−*
*y w*; *P[w^+^ hsp83:roX1]*	+	−
*w*; *P[w^+^ hsp83:msl2] msl3*	+/−	ND
*w^1118^*; *Dp(1;3)DC112*	+*^a^*	−*^a^*
*w^1118^*; *Dp(1;3)DC244*	−*^a^*	−*^a^*
*w^1118^*; *Dp(1;3)DC246*	+*^a^*	−*^a^*
*w^1118^*; *P[w^+^ SelG CG1840]*	++*^a^*	++*^a^*
*roX1^ex6^ roX2*; *P[w^+^ SelG CG1840]*	++*^a^*	++*^a^*

More than 100 nuclei were scored for each genotype. +, 30−50% of all fully polytenized nuclei are stained at *PoX1* and/or *PoX2*; −, no nuclei are stained at *PoX1* and/or *PoX2*; *+/−*, *<5%* of all fully polytenized nuclei are stained at *PoX1* and/or *PoX2*; *++*, >50% of all fully polytenized nuclei are stained at *PoX1* and/or *PoX2*; ND, not determined.

a Refers to frequency of POF targeting to transgene or duplicated region.

### Minimal POF recruitment element

**Figure 5:**
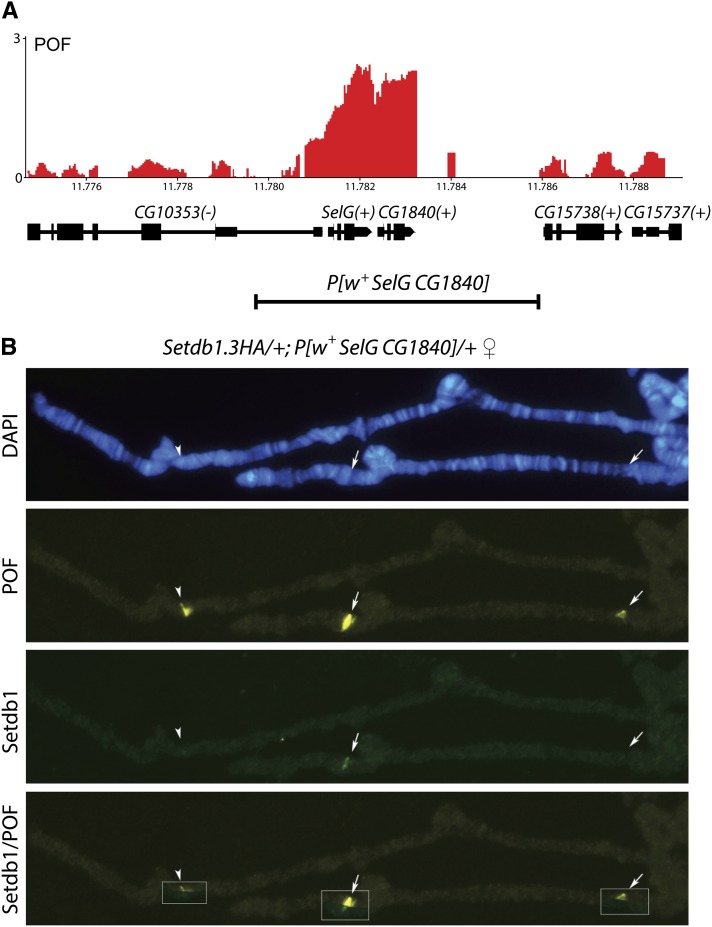
POF binds to a transgenic minimal *PoX* site. (A) High-resolution enrichment profiling at the *PoX1* site shows that POF binds to the two genes *SelG* and *CG1840*. The plots show the mean enrichment values obtained as log_2_ ChIP ratios. Numbers on the x-axis denote chromosomal position in megabases, and the y-axis shows ChIP enrichments. The genes are indicated; genes expressed from left to right are indicated by (+) and genes expressed in the opposite direction are indicated by (−). The extent of the 6.3-kb transgenic construct *P[w^+^ SelG CG1840]* is indicated. (B) POF (yellow) and Setdb1 (green) immunostaining of a *Setdb1.3HA/+*; *P[w^+^ SelG CG1840] /+* female shows that POF and Setdb1 target both endogenous *PoX1* and *PoX2* (indicated by arrows) as well as the heterozygous transgene *P[w^+^ SelG CG1840]* inserted at position 3L:65B (arrowhead). The boxes in the Setdb1/HP1 row show the combined image. Setdb1 is visualized using the Setdb1.3HA hemagglutinin tagged Setdb1 and an anti-HA antibody.

In contrast to the targeting of the MSL-complex, no high-affinity sites have previously been identified for POF binding. If the entire polytenized region of the 4^th^ chromosome (~1.2 Mb) is translocated, it will not recruit POF if not under “heterochromatic pressure” (Johansson *et al.* 2007a). The *PoX1* and *PoX2* regions were therefore of interest as nonheterochromatic targets for POF binding, and we decided to dissect these regions further. First we tested the following duplications of the *PoX1* and *PoX2* loci: *Dp(1;3)DC112* (92 kb covering *PoX1*), *Dp(1;3)DC244* (87 kb covering the three genes *Ck2β*, *Hsc70-3*, *CG1578* in *PoX2*), and *Dp(1;3)DC246* (102 kb covering the two genes *SelG* and *CG1840* in *PoX2*). These three duplications are all inserted in the same *attP* docking site in genomic region 3L:65B2 (Venken *et al.* 2010). Importantly, these duplications do not include *roX1* or *roX2* and will therefore reveal whether proximity to *roX* genes is required for targeting. In two of these duplications, *DC112* and *DC246*, POF clearly targeted the duplicated regions at frequencies similar to those of the endogenous loci (30−50%). In contrast, in *Dp(1;3)DC244*, POF was not targeted to the duplicated region. In agreement with its behavior towards the endogenous loci, POF targeting to *DC112* and *DC246* was also to a large extent dependent on *roX*, *i.e.*, POF was targeted to the duplicated region in <5% of nuclei in both *roX1 roX2*; *Dp(1;3)DC112* and *roX1 roX2*; *Dp(1;3)DC246* females (Table 1). To narrow down the region further, we tested flies transgenic for single genes or small regions. A 6-kb transgene including the genomic region covering the *Ck2β* gene, which has previously been described (Jauch *et al.* 2002), was tested for POF targeting. No targeting to the *P[w^+^ CkIIβ gDNA]* transgenic locus was detected, a result consistent with the lack of targeting in the duplication *Dp(1;3)DC244*, which covers *Ck2β*. We next cloned and generated transgenic flies with a 6-kb genomic region including the *SelG* and *CG1840* genes targeted by POF in the *PoX2* locus and in *Dp(1;3)DC246* (Figure 5A). In *P[w^+^ SelG CG1840]* females we observed consistently strong targeting to the transgenic insert (Figure 5B). The targeting appears stronger than that at the endogenous site, since targeting is seen both in males and females and at a higher frequency (>50%) than that to the endogenous *PoX* loci (Table 1).

Furthermore, targeting of POF to *P[w^+^ SelG CG1840]* in males was not abolished in the *roX1 roX2* mutant background. In agreement with findings that we have previously reported for the 4^th^ chromosome (Lundberg *et al.* 2013), POF and Setdb1 also colocalize on *PoX* loci and on the targeted *P[w^+^ SelG CG1840]* transgene (Figure 5B). We conclude that the 6-kb region inserted in *P[w^+^ SelG CG1840]* is a uniquely strong high-affinity recruitment site for POF targeting and is targeted independent of the genomic environment. The targeting of POF is also linked to the targeting by Setdb1 and HP1.

## Discussion

Two chromosome specific targeting systems have been described in *Drosophila melanogaster*: the MSL-complex, which specifically targets the male X-chromosome, and POF, which targets the 4^th^ chromosome in both males and females (Larsson and Meller 2006; Stenberg and Larsson 2011). Both the MSL-complex and POF target active genes and stimulate gene expression. The two systems stimulate gene expression levels to a comparable extent (Hamada *et al.* 2005; Johansson *et al.* 2007a; Deng *et al.* 2009; Stenberg *et al.* 2009; Zhang *et al.* 2010). High-affinity sites have been characterized for the MSL-complex and there are several published examples of short regions, including the *roX1* and *roX2* loci, that are capable of recruiting this complex when presented as transgenes (Kelley *et al.* 1999; Oh *et al.* 2004; Dahlsveen *et al.* 2006; Kind and Akhtar 2007; Alekseyenko *et al.* 2008). In contrast, until now no high-affinity sites for POF targeting have been identified. Translocated 4^th^ chromosomes will not be targeted by POF, unless the proximal heterochromatic region is present and under conditions that favor heterochromatin formation (Johansson *et al.* 2007a). The characterization of POF targeting to *PoX1* and *PoX2* in females thus provides a unique opportunity to study the targeting of POF to a nonheterochromatic target and to further our understanding of the evolution of these two targeting systems.

### POF binding to *PoX1* and *PoX2*

Considering the evolutionary relationship between POF and the MSL-complex, it was intriguing to find POF targeting to two distinct regions on the X-chromosome, *i.e.*, X:3E and X:10E-F. The apparent spreading of POF targeting in these two regions (resembling the spreading of the MSL-complex when it is targeted to *roX* transgenes) and the close location of these regions to *roX1* and *roX2* suggested a link with the MSL-complex and dosage compensation. It has been hypothesized that POF originated as a dosage compensation system, since POF targets the male X-chromosome in, for example, *D. busckii* and *D. ananassae* and in those species POF colocalizes with H4K16ac and the MSL-complex, respectively (Larsson *et al.* 2004; Stenberg and Larsson 2011). However, the targeting of POF to endogenous *PoX1* and *PoX2* in *D. melanogaster* is restricted to females. This sex-specific targeting is not caused by sex-specific expression of the targeted genes, since comparable expression levels of *RE64691* as well as *SelG* and *CG1840* are consistently found in male and female salivary glands.

### *PoX* targeting depends on *roX* expression

Not only are the two targeted loci, *PoX1* and *PoX2*, located in close proximity to *roX1* and *roX2*, the targeting is also largely dependent on *roX*, because losses of *roX1* alone or of *roX1* and *roX2* cause a clear decrease in the frequency of *PoX* targeting. Importantly, in all *roX* mutant conditions tested, we never found a complete loss of POF binding to the *PoX* sites. Therefore, *roX* is not absolutely required for *PoX* targeting but rather it enhances or stabilizes the interaction. The dependence of targeting on *roX* is not caused by the close proximity of the *PoX* loci to the corresponding *roX* loci, because in the duplications tested the *PoX1* and *PoX2* are located on another chromosome, *i.e.* chromosome arm 3L, and the *roX* genes are not included in the duplicated region. Despite this, two of the duplications show targeting by POF, comparable to that to the endogenous loci. Furthermore, targeting to these transgenic regions was found to be largely dependent on *roX*, which indicates that *roX* can act in *trans* to enhance or stabilize POF targeting. The most parsimonious model to explain these observations is that it is the *roX* ncRNA species that enhance or stabilize targeting of POF to these non-chromosome 4 targets. This model is supported by the fact that *roX2* overexpression seems to further increase the frequency of targeting. However, it should be stressed that endogenous *roX* expression in females is reported to be at very low levels or absent. In females, *roX1* RNA has been reported in early embryos but it appears to be lost midway through embryogenesis, whereas in males expression is maintained through adulthood (Meller *et al.* 1997; Meller 2003). *roX2* RNA first appears a few hours after *roX1*, but only in male embryos (Meller 2003).

### Identification of a high-affinity target for POF

No high-affinity sites for POF targeting have previously been identified (Johansson *et al.* 2007a). It therefore came as a surprise to us that a short (6-kb) region from *PoX2* functions as a strong ectopic target for POF in both males and females. The nonsex-specific targeting of POF to the *P[w^+^ SelG CG1840]* transgene, in contrast to *Dp(1;3)DC246* and endogenous *PoX*, may be explained by a competition of targeting between POF and the MSL-complex in males. This competition will be more pronounced at the endogenous *PoX* sites and the duplications as these are also targets for the MSL-complex in males. This finding is supported by the fact that on polytene chromosomes, *Dp(1;3)DC246* is targeted by MSL-complex in males while the *P[w^+^ SelG CG1840]* transgene is not targeted (results not shown). A competition in targeting is also supported by the reduction frequency of *PoX1* and *PoX2* targeting by POF observed in females expressing a partial MSL-complex, *i.e.* *w*; *P[w^+^ hsp83:msl2] msl3* females (Table 1). It is important to note that the targeting of POF to the *P[w^+^ SelG CG1840]* transgene was not caused by genomic location of this transgene since the same *attP* docking site (3L:65B2) was used as for the duplications of the *PoX1* and *PoX2* loci. The lack of targeting of POF to translocated parts of the 4^th^ chromosome and the strong targeting to the *PoX2* transgene suggest that the *PoX* regions may be POF targets that are functionally separable from the 4^th^ chromosome genes. Since both Setdb1 and HP1a are detected on the transgene, it appears likely that POF recruitment leads to, or is connected with, the formation of GREEN (HP1a and H3K9me enriched) chromatin structure (Filion *et al.* 2010).

The targeting of POF to the 4^th^ chromosome depends on its well-characterized heterochromatic nature and on the presence of HP1a and Setdb1. It is therefore important to note that links between the MSL-complex, *roX1* and *roX2* and heterochromatic regions have been reported previously, though they remain to be understood. It is known that in *roX1 roX2* mutant males, the MSL-complex is still detected on the X-chromosome, albeit at a reduced number of sites, but binding is also found in the chromocenter and at a few reproducible sites on the 4^th^ chromosome (Meller and Rattner 2002; Deng and Meller 2006; Johansson *et al.* 2011). In contrast to the X-chromosome, where the MSL-complex is believed to stimulate gene expression, loss of *roX* RNA reduces expression from genes located in the chromocenter and on the 4^th^ chromosome (Deng *et al.* 2009). It has been suggested that *roX* RNAs participate in two distinct regulatory systems, the dosage compensation system and a system for the modulation of heterochromatin (Deng *et al.* 2009). Although the mechanism by which *roX* RNAs enhance binding of POF to *PoX* loci remains elusive, the observation supports a model linking dosage compensation to modulation of heterochromatin. Additional factors supporting a model linking heterochromatin to dosage compensation are the proposed binding of HP1a to the male X-chromosome (de Wit *et al.* 2005) and the fact that a reduction in the histone H3S10 kinase JIL-1 results in the spreading of heterochromatic markers (such as H3K9me2 and HP1a) along the chromosome arms, with the most marked increase taking place on the X-chromosomes (Zhang *et al.* 2006). The JIL1 kinase, which is believed to counteract heterochromatin formation, is highly enriched on the male X-chromosome (Jin *et al.* 1999, 2000; Regnard *et al.* 2011) and is reported to be loosely attached to the MSL-complex (Jin *et al.* 2000; Wang *et al.* 2013). It is noteworthy that POF, which targets genes in a heterochromatic environment, *i.e.*, on the 4^th^ chromosome, has an intrinsic ability to target the male X-chromosome, as seen in, *e.g.*, *D. ananassae*, and the targeting to X-chromosome sites reported here is dependent on *roX* RNAs. At the same time the MSL-complex, which binds to and stimulates expression of genes on the male X-chromosome, has an intrinsic ability to target heterochromatin as seen in the *roX1 roX2* mutant background. The link between these two systems is intriguing and promises to increase our understanding of balanced gene expression.

High-affinity targeting to the *PoX1* and *PoX2* loci therefore provides a novel system for further studies on targeting mechanisms involved in chromosome-wide gene regulation, the evolutionary relationship between POF and dosage compensation and the evolution of balanced gene expression, and the results favor a model involving not only the X-chromosome but also balance to heterochromatin.
